# Eight-year tillage in black soil, effects on soil aggregates, and carbon and nitrogen stock

**DOI:** 10.1038/s41598-023-35512-x

**Published:** 2023-05-23

**Authors:** Ling Wang, Shengjie Qi, Wenfang Gao, Yang Luo, Yunpeng Hou, Yao Liang, Hongbing Zheng, Shuimei Zhang, Ruiping Li, Meng Wang, Jinyu Zheng, Zhiwei Gao

**Affiliations:** 1 Institute of Agricultural Quality Standard and Testing Technology, Jilin Academy of Agricultural Sciences, Changchun, 130033 China; 2 Institute of Agricultural Resources and Environment Research, Jilin Academy of Agricultural Sciences, Changchun, 130033 People’s Republic of China; 3 College of Life Sciences, Tianjin Normal University, Tianjin, 300387 People’s Republic of China

**Keywords:** Agroecology, Boreal ecology, Restoration ecology, Agroecology, Boreal ecology, Restoration ecology

## Abstract

The effects of different tillage management practices on the soil aggregates, soil carbon stock (STCS), and soil nitrogen stock (STNS) are key issues in agricultural research. We conducted an 8-year field experiment to evaluate the effects of different tillage methods: stubble cleaning and ridging (CK), no-tillage with stubble retention (NT), plow tillage (PT), and width lines (WL) on soil aggregates, STCS, and STNS in the black soil corn continuous cropping area of Northeast China. Different tillage methods predominantly affected the soil aggregates in the 2–0.25 mm and 0.25–0.053 mm size classes. The PT methods increased the proportion of macroaggregates and improved the quality of the soil aggregates. PT methods significantly increased the soil organic carbon content at the 0–30 cm layer by changing the number of soil macroaggregates. The PT practices are better strategies for enhancing soil carbon sinks, and the WL method increased the total amount of N in the soil pool. Our results suggest that the PT and WL methods are the best strategies for improving the quality of soil aggregates and preventing/reducing depletion of soil C and N in a black soil area of Northeast China.

## Introduction

Globally, black soil is an extremely precious natural fertile soil resource. In agricultural ecosystems, soil is a basic condition for crop growth and development as well as the material basis for agricultural development. The physical and chemical properties of soil is the basis for stable and high crop yields^1^. However, the physical and chemical properties of soil are extremely vulnerable to tillage management practices^2,3^. Therefore, the study of conservation tillage in black soil areas is important for sustainable development of agroecosystems based on this soil type.

If soil is analogous to the earth’s skin, soil organic carbon (SOC) is analogous to a protein in the skin^4^. The stock of SOC reflects the ability of the soil to intercept carbon. SOC is at the core of soil nutrient transformation, as it directly affects the maintenance and improvement of soil fertility^5,6^. Therefore, SOC is a key indicator of soil quality^7^. The chemical properties of SOC determine its charge and complexing ability, and they affect the decomposition rate of soil aggregates. SOC also affects the number and distribution of soil aggregates^8^. Soil aggregates are an important soil component, and their formation and stability play key roles in soil structure and carbon sequestration capacity^9^. Therefore, improving the stability of soil aggregates and organic carbon content is beneficial for regulating soil water, fertilizer, gas, heat, and the supply of crop nutrients. However, agricultural activities such as tillage and fertilization can easily destroy most of the soil aggregate structure^10,11^. Maintaining proper soil organic matter content and water stability is essential in soil management strategies for improving soil quality and maintaining sustainable development of agricultural ecosystems^12,13^.

Tillage occupies a vital position in agricultural production and has a significant impact on the physical and chemical properties of soils, but the effects of different tillage methods on the physical and chemical properties of soils are not the same^14,15^. Conventional tillage methods substantially disturb the soil surface structure and have a negative impact on the physical and chemical properties of agricultural soil^16^. Conservation tillage measures such as less-tillage or no-tillage can improve soil health parameters (physical, biological, and chemical) in crop and soil management systems and reduce farming costs; they are usually regarded as sustainable tillage management practices^17^. Therefore, suitable tillage methods, such as conservation tillage, can improve soil physical and chemical properties, which play important roles in soil productivity and crop yields, thereby improving food security worldwide^18,19^. Studies have shown that conservation tillage significantly changes the physical and chemical properties of the 0–20 cm soil layer, especially by facilitating an increase in SOC content^20,21^. Zhou et al. (2009) improved the structure of the plow layer of the soil by implementing different degrees of conservation tillage, creating a soil environment conducive to the extension of crop roots and the growth and development of crops, thereby increasing crop yields^22^. Ji et al. (2013) found that plow tillage is beneficial for breaking the bottom of the plow, reducing soil compactness, and creating a suitable soil environment for increased crop growth and yield^23^; it facilitates growth and development of crop roots and absorption of soil nutrients^24,25^. Monneveux et al. (2006) have asserted that no-tillage with stubble retention increases SOC and soil nitrogen content, but their study shows a certain degree of yield reduction under humid climate conditions^26^. Fabrizzi et al. (2005) found no difference in yield between less-tillage and no-tillage treatments under fertilization; however, under no fertilization, the yield from the less-tillage treatment exceeded that from the no-tillage treatment, with no significant effect on the soil physical and chemical properties^27^.

In this study, we conducted an 8-year field experiment to evaluate the effects of different tillage practices on soil aggregates and carbon and nitrogen stock in a black soil area of Northeast China. The objectives of this study were to determine: (1) the effects of different tillage practices on soil aggregation, (2) the effects of different tillage practices on soil carbon and nitrogen stock, and (3) Which tillage practice can improvement of the quality of soil aggregates and prevents/ reduces depletion of soil carbon and nitrogen.

## Results

### Tillage method effects on water-stable soil aggregates

#### Distribution characteristics of water-stable soil aggregates

**Figure 1:**
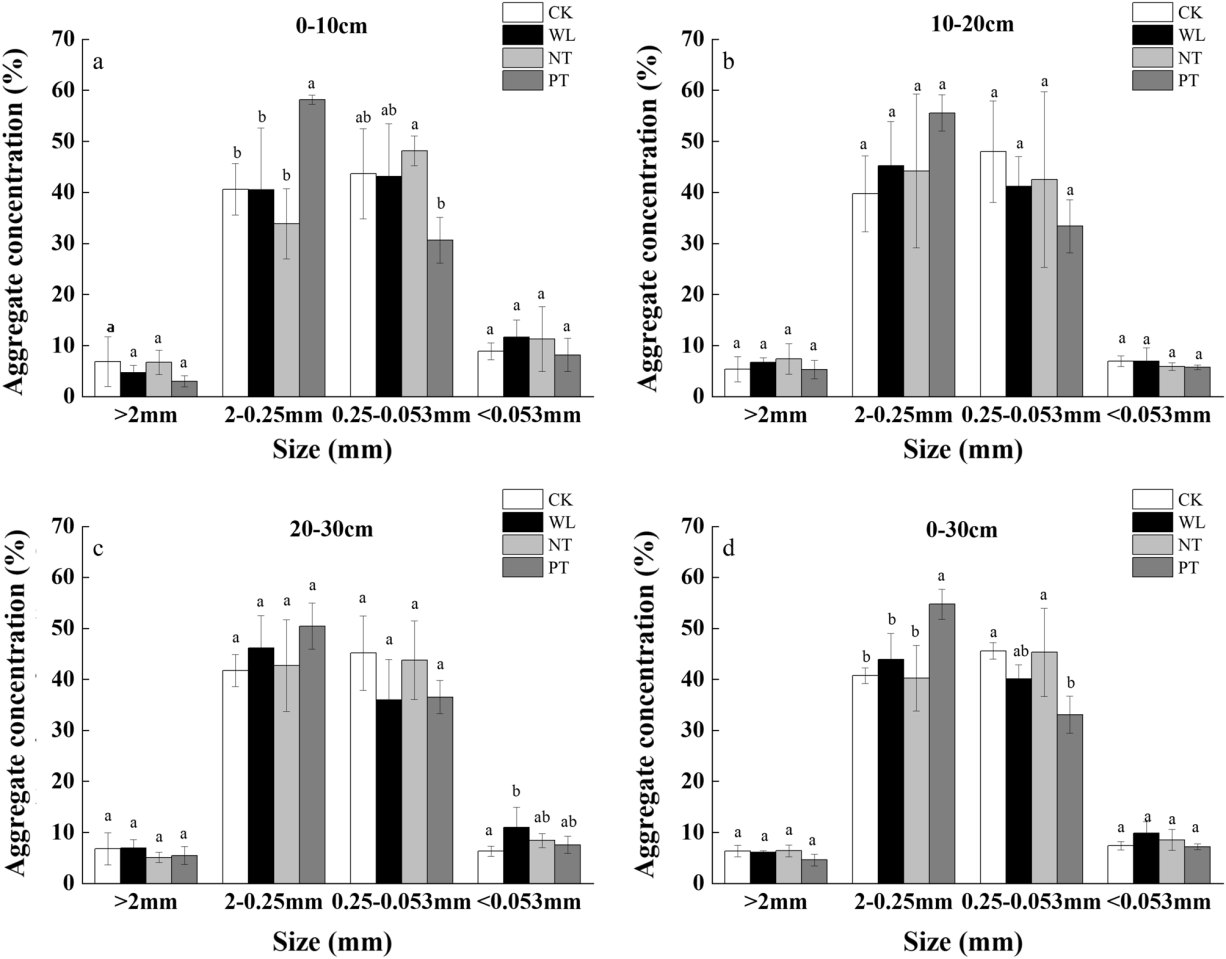
Water-stable soil aggregate distribution in the 0–10 cm (**a**), 10–20 cm (**b**), 20–30 cm (**c**), and 0–30 cm (**d**) layers of the soil under different tillage treatments applied during a 8-year continuous cropping experiment conducted in a black soil area in Northeast China. *Zea mays* L. ‘Zhengdan 958’ was cultivated for the duration of the experiment (2011–2019). CK, stubble cleaning and ridging; WL, width lines; NT, no-tillage with stubble retention; PT, plow tillage. The same letter above each bar indicates that the data are not significantly different (*P* > 0.05).

As shown in Fig. 1, the > 0.25 mm soil aggregates dominated the soil throughout the 0–30 cm layer, accounting for 45.10–61.16% of the total aggregates. The main difference in the distribution of soil aggregates was observed in the 0.25–2 mm and the 0.053–0.25 mm size classes, which was mainly manifested in the 0–10 cm soil depths. In the 0–10 cm soil depth, compared to the CK treatment, the content of the 0.25–2 mm aggregate size classes in WL and NT treatments reduced by 0.30% and 16.69%, respectively (*P* < 0.05). For the PT treatment, the content of the 0.25–2 mm size class was significantly higher than that of the CK treatment by 43.16% (*P* < 0.05).

#### Stability characteristics of water-stable soil aggregates

The stability of water-stable soil aggregates, as measured by the MWD, GMD, and E_LT_ values varied with soil layer under different tillage treatments (Table 1). In the 0–30 cm layer, compared to the other treatments, the GMD and MWD values under the PT treatment increased by 25.72% and 15.93%, respectively (*P* < 0.05); whereas the E_LT_ value under the PT treatment declined by 23.13% (*P* < 0.05).

**Table 1:** Distribution of soil aggregates by geometric mean diameter (GMD), mean weight diameter (MWD), and unstable aggregate index (E_LT_) in different layers of soil under four tillage treatments applied during a 8-year continuous cropping experiment conducted in a black soil area in Northeast China. *Zea mays* L. ‘Zhengdan 958’ was cultivated for the duration of the experiment (2011–2019).

Parameters	Treat	Soil depth (cm)			
		0–10	10–20	20–30	0–30
GMD	CK	0.38 ± 0.10^ab^	0.36 ± 0.08^a^	0.39 ± 0.05^a^	0.38 ± 0.021^b^
	WL	0.35 ± 0.11^ab^	0.42 ± 0.08^a^	0.41 ± 0.07^a^	0.39 ± 0.044^b^
	NT	0.32 ± 0.08^b^	0.43 ± 0.13^a^	0.38 ± 0.07^a^	0.38 ± 0.056^b^
	PT	0.48 ± 0.02^a^	0.50 ± 0.06^a^	0.45 ± 0.05^a^	0.48 ± 0.038^a^
MWD	CK	0.66 ± 0.14^a^	0.63 ± 0.12^a^	0.68 ± 0.09^a^	0.66 ± 0.030^b^
	WL	0.62 ± 0.15^a^	0.71 ± 0.08^a^	0.72 ± 0.08^a^	0.68 ± 0.046^ab^
	NT	0.59 ± 0.12^a^	0.71 ± 0.18^a^	0.65 ± 0.09^a^	0.65 ± 0.077^b^
	PT	0.77 ± 0.03^a^	0.78 ± 0.07^a^	0.74 ± 0.06^a^	0.76 ± 0.048^a^
E_LT_	CK	52.52 ± 9.88^ab^	54.90 ± 9.88^a^	51.46 ± 6.26^a^	52.93 ± 2.20^a^
	WL	54.81 ± 13.87^ab^	48.11 ± 8.27^a^	46.93 ± 7.40^a^	49.94 ± 4.81^a^
	NT	59.43 ± 9.24^a^	48.42 ± 19.73^a^	52.18 ± 8.96^a^	53.34 ± 7.17^a^
	PT	38.84 ± 1.80^b^	39.13 ± 5.30^a^	44.06 ± 4.95^a^	40.68 ± 3.95^b^

Data are represented as the mean ± SD. The same letter indicates that the data are not significantly different (*P* > 0.05). CK, stubble cleaning and ridging; WL, width lines; NT, no-tillage with stubble retention; PT, plow tillage.

#### Tillage method effects on soil organic carbon (SOC) and soil total nitrogen (TN)

**Figure 2:**
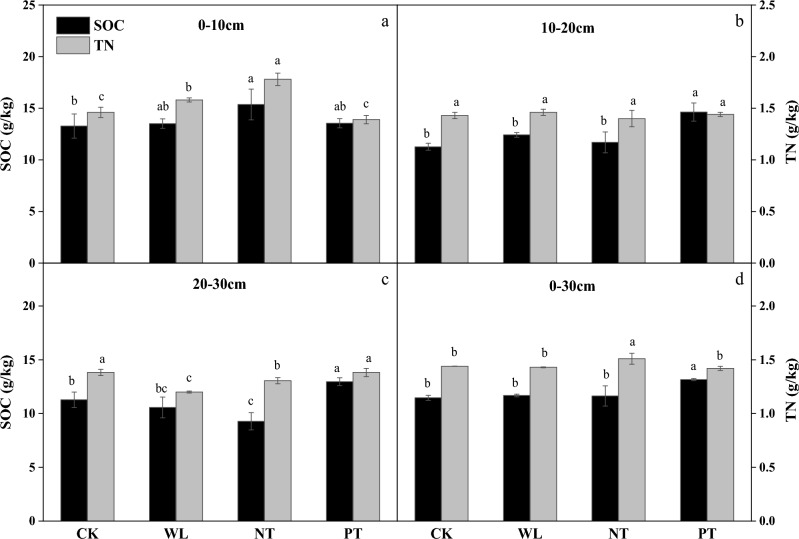
Soil organic carbon (SOC) and total nitrogen (TN) content in the 0–10 cm (**a**), 10–20 cm (**b**), 20–30 cm (**c**), and 0–30 cm (**d**) layers of the soil under different tillage treatments applied during a 8-year continuous cropping experiment conducted in a black soil area in Northeast China. *Zea mays* L. ‘Zhengdan 958’ was cultivated for the duration of the experiment (2011–2019). CK, stubble cleaning and ridging; WL, width lines; NT, no-tillage with stubble retention; PT, plow tillage. The same letter above each bar indicates that the data are not significantly different (*P* > 0.05).

As shown in Fig. 2, in the 0–10 depth, compared to CK treatment, SOC significantly increased in NT treatment (*P* < 0.05), whereas TN markedly rose in WL and NT treatments, respectively (*P* < 0.05). In the 10–20 and 20–30 cm depth, compared to other treatments, the PT treatment significantly increased SOC (*P* < 0.05). Overall, in the 0–30 cm depth, the PT treatment had the highest SOC content (13.71 g kg^−1^) in all treatments, whereas the NT treatment had the highest TN content (1.51 g kg^−1^) among all treatments (*P* < 0.05).

#### Tillage method effects on soil total carbon stock and soil total nitrogen stock

**Figure 3:**
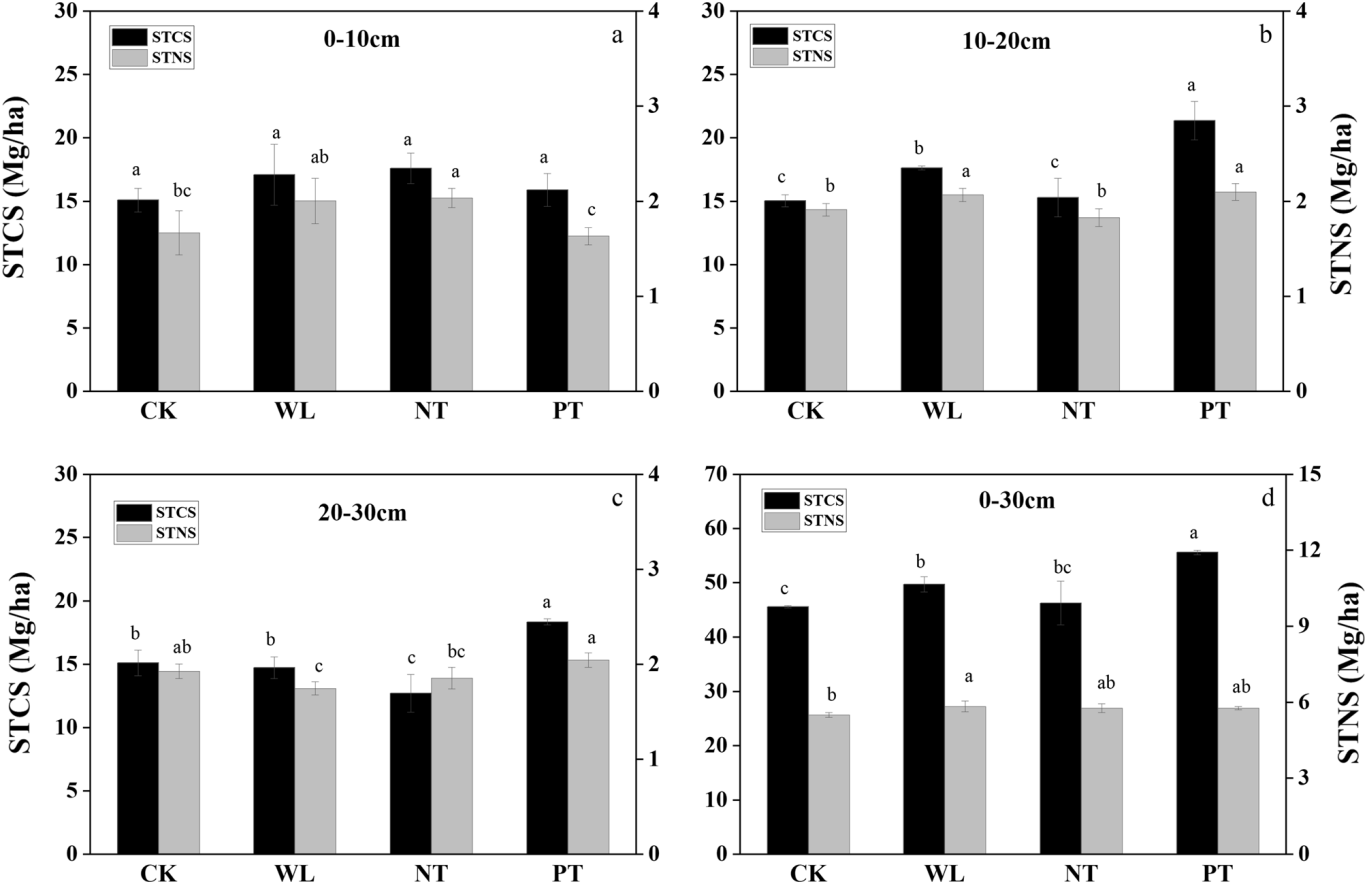
Soil carbon stock (STCS) and soil nitrogen stock (STNS) in the 0–10 cm (**a**), 10–20 cm (**b**), 20–30 cm (**c**), and 0–30 cm (**d**) layers of the soil under different treatments applied during an 8-year continuous cropping experiment conducted in a black soil area in Northeast China. *Zea mays* L. ‘Zhengdan 958’ was cultivated for the duration of the experiment (2011–2019). CK, stubble cleaning and ridging; WL, width lines; NT, no-tillage with stubble retention; PT, plow tillage. The same letter above each bar indicates that the data are not significantly different (*P* > 0.05).

The STCS and STNS at different soil depths under different tillage management practices are shown in Fig. 3. In the 0–10 cm depth, compared to CK and PT treatments, NT treatment significantly increased the STNS (*P* < 0.05). In the 10–20 cm depth, compared to CK treatment, STCS and STNS markedly rose under PT treatment by 41.93% and 9.74%, respectively (*P* < 0.05). Compared to the CK treatment, the STCS under the WL treatments rose by 17.11% (*P* < 0.05). In the 20–30 cm depth, compared to other treatments, PT treatment markedly increased STCS (*P* < 0.05). In general, in the 0–30 cm depth, the PT treatment had the highest STCS (55.6 Mg ha^−1^) among all treatments (*P* < 0.05). The STNS under WL treatment was significantly higher than that under CK (*P* < 0.05).

### Soil aggregate, soil organic carbon, and total nitrogen correlations

**Figure 4:**
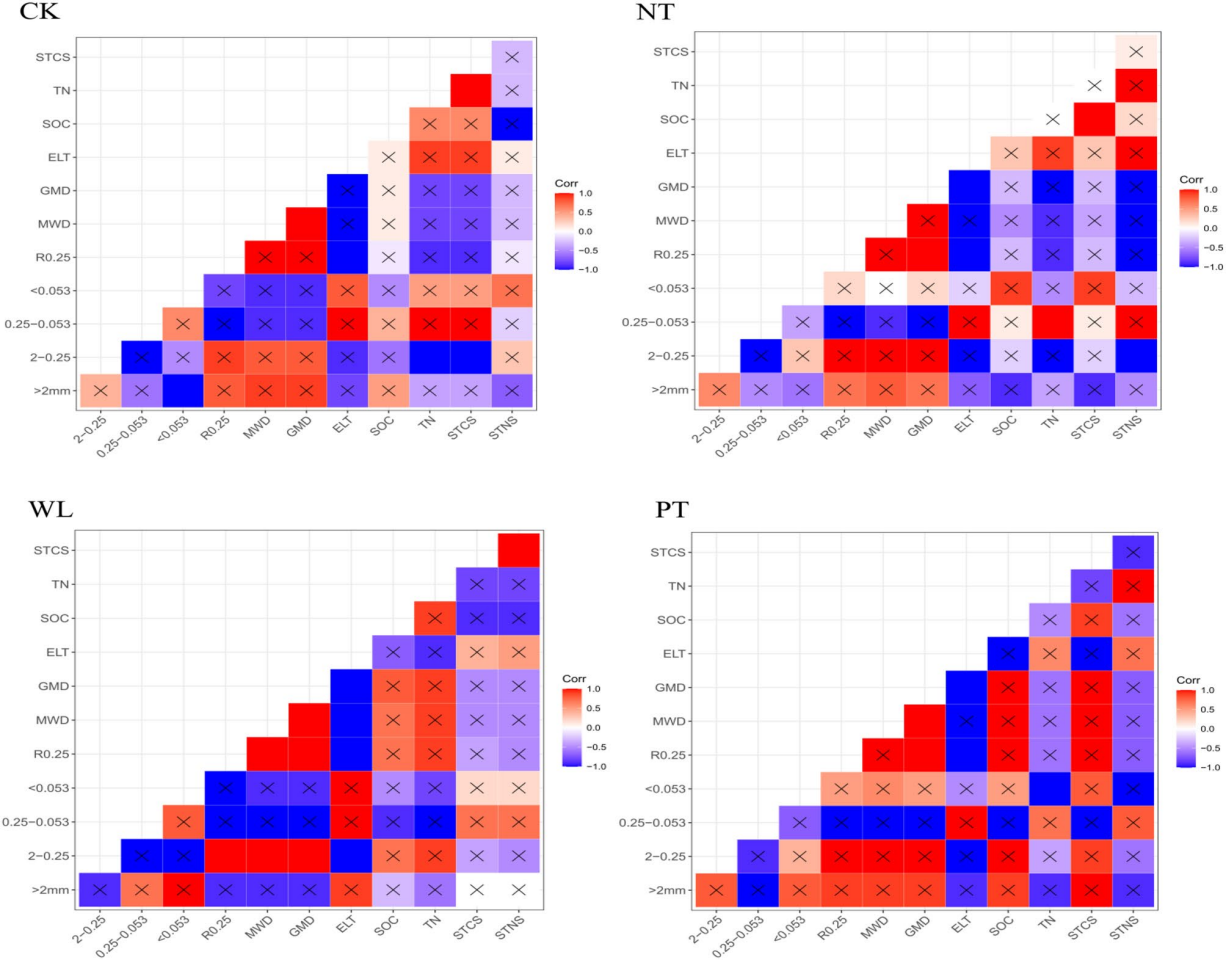
Correlation of soil aggregate characteristics and the amounts of soil carbon and soil nitrogen in the 0–30 cm layer of the soil under different tillage treatments applied during an 8-year continuous cropping experiment conducted in a black soil area in Northeast China. *Zea mays* L. ‘Zhengdan 958’ was cultivated for the duration of the experiment (2011 to 2019). CK, stubble cleaning and ridging; WL, width lines; NT, no-tillage with stubble retention; PT, plow tillage. The distribution of water-stable soil aggregates is > 2 mm, 2–0.25 mm, 0.25–0.053 mm, and < 0.053 mm. R_0.25_, aggregates > 0.25 mm; MWD, mean weight diameter; GMD, geometric mean diameter, E_LT_, unstable aggregate index; SOC, soil organic carbon; TN, total nitrogen; STCS, soil carbon stock; STNS, soil nitrogen stock. The × indicates that the data are significantly different (*P* < 0.05).

Correlations among the soil aggregate index, SOC, TN, STCS, and STNS showed significant differences under different tillage management practices at 0–30 cm soil layer (Fig. 4). Overall, in all treatments, the amount of aggregates in the 2–0.25 mm size class showed a strong positive correlation with R_0.25_, MWD, and GMD, and a strong negative correlation with E_LT_ (*P* < 0.05). However,the aggregates of the 0.25–0.053 mm size class showed a opposite trend (*P* < 0.05).

## Discussion

In an 8-year positioning experiment, we explored the comprehensive effects of soil physical and chemical properties under continuous cropping of corn cultivated using the tillage methods of stubble cleaning and ridging (CK), width lines (WL), no-tillage with stubble retention (NT), and plow tillage (PT). We considered the effects of these tillage methods on soil aggregate properties, SOC, TN, STCS, and STNS.

### Tillage method effects on water-stable soil aggregates

Soil aggregates are the foundation of soil structure, material, energy protection, and metabolism^6^. The diameter, quantity, quality, and percentage of soil aggregates can reflect soil nutrient cycling and the ability of soil to retain water and fertilizer. The stability of aggregates is crucial for the function of farmland ecosystems^28,29^. The stability of soil aggregates determines their ability to resist exogenous effects and to remain stable when exposed to changes in the external environment^30^. In addition, aggregates are closely related to soil erodibility and play an important role in maintaining the stability of the soil structure. The quantity and quality of soil aggregates directly affect soil properties and SOC sequestration^31,32^. Our results showed that 8 consecutive years under different tillage management practices significantly changed the properties of the soil aggregates. The main difference in the distribution of soil aggregates was observed in the 2–0.25 mm and the 0.25–0.053 mm size classes. It is noteworthy that in the 0–10, 10–20, 20–30, and the 0–30 cm soil layers, the relative content of aggregates in the 2–0.25 mm size class was higher under the PT treatment compared to that under other treatments; whereas the relative content of aggregates in the 0.25–0.053 mm size class under the PT treatment was lower. These findings suggest that plow tillage created a soil environment suitable for STCS and plant growth.

Although our findings showed that the WL and PT treatments caused physical destruction of the soil aggregates by plowing, we observed that the quality of the aggregates in WL and PT treatments was better compared to that in the CK and NT treatment soils. There may be two reasons for this. First, although plowing potentially fragments soil aggregates, it also transports plant residues (stubble and roots) downward, facilitating the exchange of organic matter between the topsoil and deeper soil layers. Second, under the WL and PT treatments, stubble and roots are retained in the soil, and they potentially enhance the formation of soil macroaggregates^33^. Some studies have shown that the addition of organic matter can improve the soil aggregation process, because organic matter (such as cellulose) acts as a binder for soil aggregates^34,35^. Organic matter in the soil can create contact points, provide nutrients for microorganisms, and promote the formation of soil aggregates^36,37^.

The values of MWD and GMD can be used to assess soil structural stability^38^. The E_LT_ value is the ratio of the mass of aggregates in the < 0.25 mm size class to the total aggregate mass; it can be used to evaluate soil erosion resistance and stability as well as to reflect the quality of the soil structure^39^. Under the PT treatment, we found that the MWD and GMD values of the 0–30 cm soil layers were higher compared to those of the other three treatments, and the E_LT_ value was lower compared to that under the other three treatments. These findings were consistent with the distribution of soil aggregates that we observed; they indicated that the condition of soil agglomeration under the PT treatment was better overall compared to that under other treatments, and the PT treatment enhanced soil erosion resistance and stability. Therefore, it appears that the addition of organic residues under the PT treatment improved the soil structure in the 0–30 cm through plowing, and it increased the stability of the soil aggregates.

### Tillage method effects on soil organic carbon and total nitrogen

As the core of soil quality and function, SOC is an important indicator of soil quality and health^6^. The SOC content can directly affect soil fertility and crop yield, which are greatly affected by the formation and stability of water-stable aggregate structure^35,40,41^. Increasing SOC accumulation can indirectly affect crop yield by maintaining soil structure and regulating soil microbial activity^42^. We found that in the 0–30 cm layer, the SOC content and STCS under the PT treatment were higher compared to those under other treatments. Some studies have shown that soil aggregates are an important physical dimension of soil protection, and nearly 90% of organic carbon sequestration occurs in soil aggregates^37,43,44^. Macroaggregates can provide physical protection to prevent microorganisms from mineralizing SOC. Because the size, quantity, and composition of soil aggregates are sensitive to changes in SOC, aggregates can potentially be used as indicators of carbon sequestration under different tillage management practices^45–48^. This is consistent with our experimental findings. Our correlation analysis showed that SOC was significantly positively correlated with R_0.25_ under the PT treatment. Therefore, we suggest that the PT treatment significantly increased the SOC content in the 0–30 cm soil layer by changing the number of soil macroaggregates during the 8-year experiment, thereby enhancing soil carbon sequestration in this black soil area of Northeast China.

As important soil nutrient components, TN is the main sources of essential nutrients for plant growth. As key indicators of soil quality, STCS and STNS strongly influence soil productivity, sustainable land use, and environmental protection^49^. We found that in the 0–30 cm soil layer, N stock under the WL treatment was higher compared to that under the other three treatments. This suggests that the WL treatment significantly increased the nitrogen sinks in the soil. We speculated that the WL treatment of plowing promoted changes in the microbial community through substantial changes in the soil microenvironment. This may be related to the high C/N ratio of corn plant residues and the different decomposition rates of soil microorganisms^31,48,50,51^. However, the underlying mechanism is still unclear, and we need to further analyze the structure and function of soil microbial communities.

## Conclusions

The effects of different tillage management practices on the physical structure, STCS, and STNS of agricultural soils are key issues in current agricultural research. Our study found that plow tillage methods increased the proportion of macroaggregates, and plow tillage also increased the aggregate stability. In addition, we found that plow tillage methods significantly increased the SOC content in the 0–30 cm layer by changing the number of soil macroaggregates, thereby enhancing soil carbon sinks in the black soil under study. In addition, the width lines method increased the total amount of N in the soil pool. Therefore, we suggest that width line and plow tillage management practices in the black soil area of Northeast China are the best strategies to improve the quality of soil aggregates and prevent depletion of soil C and N according to an 8-year experiment.

## Materials and methods

### Experimental site

The study was conducted in an experimental field at the Jilin Academy of Agricultural Sciences, Gongzhuling, Jilin Province, China (43°3023″ N, 124°4833.8″ E), where we had rights of soil land use and permission to conduct our experiment. This is a typical rain-fed agricultural area, which has a mid-temperate continental monsoon climate with an annual average temperature of 4–6 °C, a frost-free period of 110–140 d, an effective accumulated temperature of 2600–3000 °C d, an annual precipitation of 450–650 mm, an annual evaporation of 1,200–1,600 mm, and annual sunshine hours of 2500–2700 h. The soil is classified as black soil (Typic Hapludoll, USDA Soil Taxonomy) with a clay loam texture (36.0% clay, 24.5% silt, and 39.5% sand). The pH of the topsoil (0-20 cm) was 6.5.

### Experimental design

The test plot is an 8-year positioning test site with flat terrain and uniform topography. The experiment was started in 2011 and has been a positioning test for 8 consecutive years. The test crop, corn variety *Zea mays* L. ‘Zhengdan 958’, has been cultivated under continuous cropping; grown each year at a density of 60,000 plants ha^−1^. A large-area comparison experimental approach was adopted, involving four tillage methods: stubble cleaning and ridging (CK), no-tillage with stubble retention (NT), plow tillage (PT), and width lines (WL). Each treatment area was 1260 m^2^ (12 ridges × 0.7 m × 150 m); the ridge spacing was 0.7 m. The annual fertilizer application rate for each treatment was N 200 kg hm^−2^, P 39.3 kg hm^−2^, and K 62.5 kg hm^−2^. All the P_2_O_5_ and K_2_O fertilizer, and 40% of the N fertilizer were applied as base fertilizer, and the remaining 60% of the N fertilizer was applied to the topsoil and 10 cm below the topsoil at the jointing stage of the corn crop. The field operations and details of each tillage method are presented in Table 2.

**Table 2:** Field operations associated with four tillage methods used during a 8-year continuous cropping experiment conducted in a black soil area in Northeast China. *Zea mays* L. ‘Zhengdan 958’ was cultivated for the duration of the experiment (2011–2019).

Tillage method	Details of field operations
Stubble cleaning and ridging (CK)	When harvesting in autumn, no high stubble is left, and the stubble is ridden and ridged in autumn to reach the sowing state
Width lines (WL)	During processing, corn is planted into wide (90 cm) and narrow (40 cm) rows. After the autumn harvest, the land is prepared into wide rows by rotary tillage to reach the sowing state
No-tillage with stubble retention (NT)	When harvesting in autumn, the ears of corn are pulled away, leaving the stubble in the field without plowing. Direct mechanized sowing between the rows with stubble takes place the following spring
Plow tillage (PT)	During processing, after the corn is harvested, the stalks are cut and pulled away, and normal turning, raking, and sowing operations are performed

### Sampling methods

Soil samples were collected using a soil sampler (diameter: 8 cm) at three depths (0–10, 10–20, and 20–30 cm) in November 2019, after the autumn harvest. Three replicates were set for each treatment. Random sampling at multiple points in the sample plot was repeated to reduce the impact of soil differences. Soil samples were collected from three points in each treatment, providing a total of 36 samples (3 plots × 3 depths × 4 treatments) for the experiment. The soil samples collected from each layer were placed into separate sterile polyethylene ziplock bags, labeled, and transported to a laboratory for analysis. Each soil sample was divided into two parts. One part was gently broken along the natural structural cracks of the undisturbed soil and dispersed into soil blocks of approximately 1 cm in size. The soil blocks were dried naturally under cool, ventilated conditions to determine soil aggregates. The other portion of each soil sample was quickly sieved (2 mm) and stored at 4 °C, and the chemical properties were determined within 1 week of field collection. All methods were performed in accordance with the relevant guidelines/regulations/legislation.

### Determination of soil physical and chemical properties

Soil agglomeration was determined using the wet sieve method. We weighed 50 g of air-dried soil and placed it evenly on the top layer of a set of three sieves with mesh dimensions of 2, 0.25, and 0.053 mm. Next, we placed the sieves in a bucket of water and completely soaked the soil for 10 min. We oscillated the sieves up and down for 10 min at an amplitude of 5 cm and a frequency of 40 times min^−1^. Thereafter, we collected the agglomerates on each soil sieve and placed them separately into aluminum boxes. The soil samples in the bucket were collected by sedimentation and centrifugation and dried at 60 °C for weighing. From the wet sieve process we obtained macroaggregates (> 2 mm and 0.25–2 mm size classes), and microaggregates (0.053–0.25 mm and < 0.053 mm size classes), which we used to determine the aggregate properties of the treatment soils. SOC and total nitrogen (TN) were measured using an elemental analyzer (PE-2400 II; PerkinElmer, Waltham, MA, USA).

### Data analysis

The properties of the soil aggregates were determined using a dataset of differently sized aggregates and the following equations:

$${\text{R}}_{0.25} = \frac{{M_{{{\text{r}} > 0.25}} }}{{{\text{M}}_{{\text{T}}} }} = 1 - \frac{{{\text{M}}_{{{\text{r}} < 0.25}} }}{{{\text{M}}_{{\text{T}}} }}$$

$$MWD = \Sigma_{i = 1}^{n} \overline{{x_{i} }} w_{i} /\Sigma_{{{\text{i}} = 1}}^{n} w_{i}$$

$$GMD = {\text{Exp}}\left[ {\Sigma_{{{\text{i}} = 1}}^{n} w_{i} \ln \overline{{x_{i} }} /\Sigma_{{{\text{i}} = 1}}^{n} w_{i} } \right]$$

$$E_{LT} = \left[ {\left( {W_{T} - W_{0.25} } \right)/W_{T} } \right] \times {1}00\%$$

where *M*_*T*_ (g) is the sum of aggregate weight; *MWD* (mm) is the mean weight diameter; *GMD* (mm) is the geometric mean diameter;* E*_*LT*_ (%) is the unstable aggregate index; $\overline{{x }_{i}}$ is the average diameter of the soil aggregates in horizon *i* (mm); *w*_*i*_ is the mass fraction of the soil aggregates in horizon *i* (%); *W*_*T*_ is the total mass of aggregates in each horizon (g); and *W*_*0.25*_ is the mass of water-stable aggregates ≥ 0.25 mm (g).

The soil total C and N stock to a depth of 30 cm was calculated as follows:

$$STC\left( N \right)S = \Sigma_{{{\text{i}} = 1}}^{n} ({1} - \theta_{i} \% ) \, \times \rho {\text{i}} \times C_{i} (N_{i} ) \times T_{i} /{1}00$$

where *STC(N)S* (kg m^−2^) is the soil total C and N stock in a profile; *θ*_*i*_ is the gravel (> 2 mm) content in horizon *i* (%); ρ_i_ is the soil bulk density in horizon i (g cm^−3^); *C*_*i*_ and *N*_*i*_ are soil total C and N content in horizon *i* (g kg^−1^); *T*_*i*_ is the thickness of horizon *i* (cm); and *n* is the number of horizons involved.

### Statistical analysis

The agricolae package in R (4.0.3; https://cran.r-project.org/), based on one-way analysis of variance, was used to explore the significant differences between soil aggregates and C- and N-related indicators under different tillage methods. The ggcorrplot and ggtheme packages in R (4.0.3) were used to evaluate the correlations between soil aggregates and C- and N-related indicators based on Pearson correlation analysis. Statistical significance was considered at *P* < 0.05.

## Data Availability

The datasets generated during and analysed during the current study are available from the corresponding author on reasonable request.
